# Spontaneous Pupillary Recovery of Urrets-Zavalia Syndrome Following Descemet’s Membrane Endothelial Keratoplasty

**Published:** 2019

**Authors:** Marco M. S. ISAC, Darren Shu Jeng TING, Trushar PATEL

**Affiliations:** 1 Department of Ophthalmology, James Cook University Hospital, Middlesbrough, UK; 2 Academic Ophthalmology, Division of Clinical Neuroscience, School of Medicine, University of Nottingham, Nottingham, UK

**Keywords:** Fuch’s Endothelial Dystrophy, Descemet Stripping Endothelial Keratoplasty, Pupil Abnormality, Urrets-Zavalia Syndrome

## Abstract

To report a case of Urrets-Zavalia syndrome (UZS) with spontaneous pupillary recovery following Descemet's membrane endothelial keratoplasty (DMEK). This was an interventional case report with literature review. A 37-year-old female phakic patient presented to our clinic with bilateral decreased vision secondary to worsening Fuch’s endothelial dystrophy. She underwent bilateral inferior peripheral iridotomies prior to undergoing left DMEK surgery under general anaesthesia. The DMEK surgery was uncomplicated but she had a large fixed and dilated left pupil on the following day despite a normal examination with a normal intraocular pressure. A diagnosis of UZS was made. The pupil remained fixed and dilated until 4 months postoperatively, which anisocoria started to improve by time. At 6 months postoperative, anisocoria had fully resolved with normal pupillary reactions and complete resolution of photopic symptoms. UZS is a rare complication of DMEK surgery and, to our best knowledge, only one case has been reported in the literature. Surgeons and patients should be aware of this potential phenomenon following uneventful DMEK surgery. Conservative measures should be considered for initial management of UZS in young patients as spontaneous recovery may sometimes ensue, as occurred in our case.

## INTRODUCTION

Fixed dilated pupillary abnormality following penetrating keratoplasty (PKP) was first described by Ramón Castroviejo Briones [1] in a patient with keratoconus. The term, Urrets-Zavalia syndrome (UZS) was then introduced in 1963 by Alberto Urrets-Zavalía in a case series of 6 patients who underwent PKP and developed postoperative iris abnormalities, predominantly of rigid dilated pupil with multiple posterior synechiae and iris atrophy [2]. The exact pathogenesis of UZS is still not fully known but intraoperative iris ischemia and postoperative rise in intraocular pressure (IOP) are the two most commonly accepted mechanisms of this rare syndrome [3]. Urrets-Zavalia syndrome has also been reported following other types of intraocular surgery including cataract [4], goniotomy [5], trabeculectomy [6] and other types of keratoplasty such as deep anterior lamellar keratoplasty (DALK) [7] and Descemet stripping automated endothelial keratoplasty (DSAEK) [8]. So far there is only one published case report in the literature on UZS following Descemet’s membrane endothelial keratoplasty (DMEK), which was associated with postoperative raised IOP [9]. Herein we presented a case of spontaneous recovery of UZS in a young phakic patient following an uneventful DMEK with normal postoperative IOP.

## CASE REPORT

This was an interventional case report with literature review. An informed written consent was obtained from the patient for all the surgical interventions described herein and publication of this case report. A 37-year-old female Caucasian patient was referred to our cornea clinic with bilateral slowly progressive deterioration of vision due to Fuch’s endothelial dystrophy (FED). Slit-lamp examination revealed bilateral extensive central corneal endothelial guttae which was particularly more pronounced in the left eye. The corrected-distance-visual-acuity (CDVA) was 20/40 in the right eye and 20/120 in the left eye, and IOP was 14 mmHg in both eyes. Otherwise the rest of the ocular examination was unremarkable. The patient underwent a left eye DMEK surgery under general anaesthesia after discussing the risks and benefits. In view of her phakic status, bilateral prophylactic inferior Nd:YAG laser peripheral iridotomies (total energy used was 4.2 millijoule (mJ) in the right eye and 18.2 mJ in the left eye) were performed prior to the DMEK surgery to reduce the risk of postoperative pupillary block from air tamponade. Topical dexamethasone was commenced immediately post-laser treatment, starting with 2 hourly for 3 days followed by 4 times a day (QID) for a week. The eyes settled down within 4 weeks with no residual intraocular inflammation and the pupils remained round and small.

The surgical technique of DMEK was adapted from previously described technique with some modifications [10, 11]. An 8 millimetre (mm) DMEK graft was manually prepared on a corneal punch block by the surgeon (T.P.). An “F” mark was stamped on the stromal surface of the donor descemet’s membrane (DM). The donor DM lenticule was then loaded into a DM implantation cartridge (Gueder AG, Germany). The central 9 mm of the host DM was scored and stripped and the DMEK graft was introduced into the anterior chamber through a 2.75 mm temporal incision. The graft was successfully unscrolled and centered before injection of 100% intracameral air to tamponade the graft against the posterior stroma. Correct orientation of the graft was confirmed with the “F” stamp. Finally, the primary corneal incision and the paracentesis were sutured with a single non-absorbable monofilament 10-0 nylon suture. The eye was dilated with a single drop of topical cyclopentolate 1% and phenylephrine 2.5% at the end of the operation. The patient was given topical dexamethasone 0.1% QID and topical chloramphenicol 0.5% QID as part of the postoperative treatment regimen.

**Figure 1:**
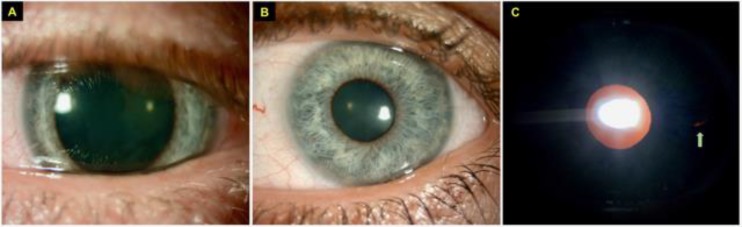
(A) Slit-lamp Photography of the Left Eye Showing Fixed, Mid-dilated Pupil One Month Following Descemet’s Membrane Endothelial Keratoplasty (DMEK) for Fuchs’ Endothelial Dystrophy, Consistent With Urrets-Zavalia Syndrome (UZS). (B-C) At 6-Month Post-DMEK, Slit-lamp Photography of the Left Eye Showing Complete Resolution of the Dilated Pupil With a Small Mid-peripheral Iris Transillumination at the Temporal Iris (Green Arrow).

During immediate postoperative period, the patient was positioned supine and in a face up posturing position for 90 minutes. Thereafter, 40% of the air was released from the anterior chamber at the slit-lamp through the single paracentesis incision. The air–fluid meniscus was seen above the inferior margin of the dilated pupil with a normal IOP. The DMEK graft was fully attached and well supported by the remaining 60% air bubble in the anterior chamber whilst supine. The patient was instructed to lie supine until the following morning. At 1-day postoperative, the patient reported severe nausea and vomiting all night such that she could not perform the instructed postoperative posturing. Despite this the DMEK graft was fully attached with only a 40% air bubble remaining in the anterior chamber. Her uncorrected-distance-visual-acuity (UDVA) in the operated left eye was 20/200. The IOP was 11 mmHg but the pupil was unresponsive to light and mid-dilated. The pupil remained enlarged at 1-week postoperative at which point a small partial (10%) localized graft detachment was noted. A successful re-bubbling of the graft was performed and a trial of topical pilocarpine 2% QID for 2 weeks was introduced to manage glare symptoms from the enlarged pupil. However, the enlarged pupil failed to improve and a diagnosis of postoperative Urrets-Zavalia syndrome (UZS) was made (Fig 1A). The patient was treated for left mild anterior uveitis at 1-month postoperative, which fully resolved after 2 weeks of increased topical dexamethasone 0.1% 6 times a day. The IOP remained normal throughout the postoperative period with no clinically evident postoperative pupillary block or posterior synechiae. The enlarged pupil improved gradually at 4 months postoperative and fully returned to normal size at 6-month postoperative with complete resolution of the symptoms of glare (Fig 1B). A small transillumination defect was noted at the mid-periphery of temporal iris (Fig 1C), which was likely resulted from the implantation cartridge during the insertion of DMEK graft. At final 9-month postoperative visit, her UDVA was 20/30 and she was receiving topical dexamethasone 0.1%.

## DISCUSSION

In this report we highlighted a case of spontaneous pupillary recovery of UZS in a young phakic patient following DMEK surgery. Urrets-Zavalia syndrome is an uncommon complication most commonly reported after PKP, with a reported incidence of 0% – 17.7%, and less commonly described following other ocular surgeries, including DALK, DSAEK and cataract surgery [3]. The increased risk of UZS after PKP compared to other surgeries is not fully understood, but various hypotheses have been proposed. These included higher risk of iris ischaemia due to compression of iris vessels against the incision edge of recipient cornea, iris injury and peripheral anterior synechiae [3]. Tuft and Buckley [12], and Figueiredo et al [13] have provided supportive evidence on the association of iris ischemia and UZS using anterior segment fluorescein angiography and indocyanine green angiography, respectively. Figueiredo et al [13] also observed delayed and reduced sectoral iris vasculature fillings corresponding to the areas of iris atrophy and impaired pupil reaction observed on clinical examination in their patients with UZS. Raised intraoperative or postoperative IOP leading to iris ischemia – albeit not a universal finding – is another major risk factor for UZS following intraocular surgeries [3].

To our best knowledge, there is only one published case of UZS after DMEK in the literature. Holtmann et al [9] reported a 74-year-old patient with FED who developed UZS after DMEK. The dilated and tonic pupil remained unchanged at 1-year postoperative, which was unresponsive to topical pilocarpine drops. It was hypothesized that UZS manifestation was related to postoperative raised IOP of 40 mmHg at day 1 and iris trauma and atrophy occurred intraoperatively. On the other hand, Arnalich-Montiel et al [14] reported a spectrum of pupillary abnormalities following DMEK, ranging from mild ovalization to mid-dilated poorly responsive pupils. Among those, 2 eyes developed poorly responsive pupils suggestive of UZS but were later found to be caused by posterior synechiae of the iris on the lens capsule. In our case, a prophylactic peripheral iridotomy was performed to prevent postoperative pupillary block and raised IOP [15]. Indeed, the IOP was normal throughout the postoperative period, postoperative vomiting might have caused a spike in IOP transiently. We also observed a minimally visible mid-peripheral iris transillumination defect which could have contributed to UZS manifestation in our case, though there was no evidence of damage to the pupillary sphincter.

Partial or, rarely, complete recovery of pupil abnormalities in patients with UZS has been reported in the literature, with a rate of around 33% [3]. Those that did not resolve were usually associated with severe iris injury or atrophy, posterior synechiae and glaukomflecken. In non-resolving or symptomatic cases, various interventions such as corneal tattooing, black diaphragm intraocular lens or iris suturing, may be attempted to address the visual symptoms. The self-limiting nature of UZS in our case was likely attributed to minimal iris injury, normal postoperative IOP and possibly the young age of our patient. Spadea et al. similarly described a regression in pupillary dilation in a young patient with UZS following DALK, though a short course of topical sympatholytic (dapiprazole 0.5%) and parasympathomimetic (pilocarpine 2%) drops was required.

## CONCLUSION

Urrets-Zavalia syndrome is a rare and likely underreported complication following DMEK surgery. We advocate prophylactic peripheral iridotomy, minimal intraoperative iris manoeuvring, careful monitoring and timely management for intraoperative/postoperative raised IOP to reduce the risk of UZS following DMEK. Conservative measures should be first considered as the initial management plan in young patients as spontaneous recovery may sometimes ensure, which was observed in our case.
